# Report of the Eastern Lunatic Asylum, Williamsburg, Va., &c.

**Published:** 1852-07

**Authors:** 


*Report of the Eastern Lunatic Asylum in the City of Williamsburg, Va.*, 1851. From Dr. JNO. M. GALT, Superintendent and Physician.

*Proceedings of the Seventh Annual Meeting of Medical Superintendents of American Institutions for the Insane, N. Y., May* 18, 1852.

THE report of Dr. Galt is full of interest to the medical man. From the tables it appears that out of 384 patients 137 became insane between the ages of 20 and 30 years, and 102 between 30

and 40,—that the admissions during the winter months were 249, during the summer months 355. Discharges during winter months 57, during summer months 83. Of 359 patients 193 had never been married. Of 361 patients, 216 of whom were males, 145 females, the number of cases of moral insanity were 30 among the males, and 33 among the females.

The Physiological Register forms an interesting feature of the report.

The next meeting of medical Superintendents is to be held in Baltimore on the second Tuesday of May, 1853. J.

